# Cryoballoon pulmonary vein isolation as first-line treatment of typical atrial flutter: long-term outcomes of the CRAFT trial

**DOI:** 10.1007/s10840-024-01786-y

**Published:** 2024-03-13

**Authors:** Peter Calvert, Wern Yew Ding, Moloy Das, Lilith Tovmassian, Muzahir H. Tayebjee, Guy Haywood, Claire A. Martin, Kim Rajappan, Matthew G. D. Bates, Ian Peter Temple, Tobias Reichlin, Zhong Chen, Richard N. Balasubramaniam, Christian Sticherling, Christina Ronayne, Nichola Clarkson, Maureen Morgan, Janet Barton, Ian Kemp, Saagar Mahida, Dhiraj Gupta

**Affiliations:** 1 Liverpool Centre for Cardiovascular Science & Liverpool Heart and Chest Hospital, Liverpool Heart & Chest Hospital NHS Foundation Trust, Liverpool, UK; 2 Department of Cardiology, The Newcastle Upon Tyne Hospitals NHS Foundation Trust, Freeman Hospital, Newcastle Upon Tyne, UK; 3 Department of Cardiology, Leeds Teaching Hospital NHS Foundation Trust, Leeds, UK; 4 Department of Cardiology, University Hospitals Plymouth NHS Foundation Trust, Plymouth, UK; 5 Department of Cardiology, Royal Papworth Hospital NHS Foundation Trust, Cambridge, UK; 6 Department of Cardiology, Oxford University Hospitals NHS Foundation Trust, Oxford, UK; 7 Department of Cardiology, South Tees Hospitals NHS Foundation Trust, James Cook University Hospital, Middlesbrough, UK; 8 Department of Cardiology, Manchester University NHS Foundation Trust, Wythenshawe Hospital, Manchester, UK; 9 Department of Cardiology, Inselspital, Bern University Hospital, University of Bern, Bern, Switzerland; 10 Department of Cardiology, Ashford and St Peter’s Hospital NHS Foundation Trust, Surrey, UK; 11 Department of Cardiology, Royal Bournemouth and Christchurch Hospital NHS Foundation Trust, Bournemouth, UK; 12 Department of Cardiology, University Hospital Basel, University of Basel, Basel, Switzerland

**Keywords:** Atrial flutter, Atrial fibrillation, Cryoballoon, Catheter ablation, Loop recorder

## Abstract

**Background:**

CRAFT was an international, multicentre, randomised controlled trial across 11 sites in the United UK and Switzerland. Given the evidence that pulmonary vein triggers may be responsible for atrial flutter (AFL) as well as atrial fibrillation (AF), we hypothesised that cryoballoon pulmonary vein isolation (PVI) would provide greater symptomatic arrhythmia reduction than cavotricuspid isthmus (CTI) ablation, whilst also reducing the subsequent burden of AF. Twelve-month outcomes were previously reported. In this study, we report the extended outcomes of the CRAFT study to 36 months.

**Methods:**

Patients with typical AFL and no evidence of AF were randomised 1:1 to cryoballoon PVI or radiofrequency CTI. All patients received an implantable loop recorder (ILR) for continuous cardiac rhythm monitoring. The primary outcome was time-to-symptomatic arrhythmia recurrence > 30 s. Secondary outcomes included time-to-first-AF episode ≥ 2 min. The composite safety outcome included death, stroke and procedural complications.

**Results:**

A total of 113 patients were randomised to cryoballoon PVI (*n* = 54) or radiofrequency CTI ablation (*n* = 59). Ninety-one patients reconsented for extended follow-up beyond 12 months. There was no difference in the primary outcome between arms, with the primary outcome occurring in 12 PVI vs 11 CTI patients (HR 0.97; 95% CI 0.43–2.20; *p* = 0.994). AF ≥ 2 min was significantly less frequent in the PVI arm, affecting 26 PVI vs 36 CTI patients (HR 0.48; 95% CI 0.29–0.79; *p* = 0.004). The composite safety outcome occurred in 5 PVI and 6 CTI patients (*p* = 0.755).

**Conclusion:**

Cryoballoon PVI shows similar efficacy to radiofrequency CTI ablation in reducing symptomatic arrhythmia recurrence in patients presenting with isolated typical AFL but significantly reduces the occurrence of subsequent AF.

**Graphical Abstract:**

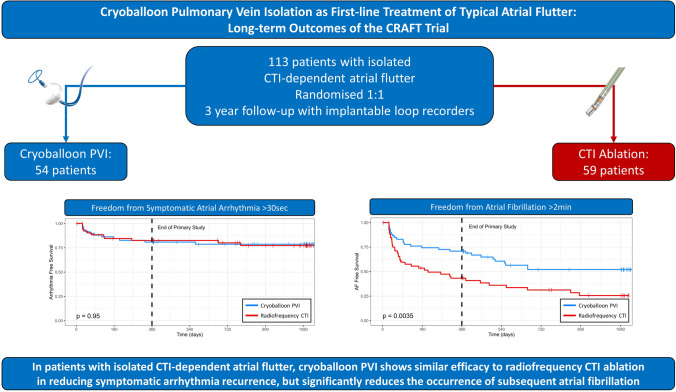

## Introduction

CRAFT (Cryoballoon Pulmonary Vein Isolation as First-Line Treatment for Typical Atrial Flutter) was an international, multi-centre, randomised controlled trial involving 11 sites across the United Kingdom and Switzerland [1]. The premise of CRAFT was that elimination of pulmonary vein (PV) triggers using cryoballoon PV isolation (PVI) would suppress typical right atrial flutter (AFL). This is based upon previous work suggesting that many patients presenting with isolated AFL have underlying atrial fibrillation (AF)—and thus triggers arising from the PVs[2–4]. Those who are prone to organising into a flutter circuit may therefore present with AFL, rather than AF.

Standard catheter ablation of the cavotricuspid isthmus (CTI)-dependent AFL involves creating a line of conduction block across the CTI, interrupting the flutter circuit. Although studies show the benefits of catheter ablation [5], this approach treats the mechanism of AFL, rather than the underlying trigger. This may explain why 50% or more patients later return with AF(6, 7). We therefore hypothesised that trigger elimination via PVI would provide better clinical outcomes in terms of symptomatic arrhythmia recurrence. As previously described, earlier studies utilised older radiofrequency (RF) technology without the benefit of contact force sensors [8]. Given the well-documented lower inter-operator variability in outcomes with cryoballoon (CB) PVI as compared to RF PVI [9] and the associated lower risk of major complications such as tamponade [10], we chose CB as the PVI tool for the CRAFT trial.

In the primary analysis [11], using continuous cardiac rhythm monitoring with implantable loop recorders (ILRs), we demonstrated similar freedom from symptomatic atrial arrhythmia regardless of whether the first-line treatment targeted the trigger (PVI), or mechanism (CTI) (HR 1.11; 95% CI 0.46–2.67; *p* = 0.82). There was no difference in safety outcomes.

In this extension study, we followed patients enrolled in CRAFT for a further 2 years, totalling 36 months. The intention was to observe the incidence of new-onset AF in addition to late AFL recurrence over the expected battery life of the ILR device.

## Methods

### Study design

The trial design and study procedures were as previously described [1, 11]. Briefly, adults aged ≥ 18 years with at least one episode of typical AFL (based on expert analysis of a 12-lead ECG) were enrolled. Participants with AF were excluded, as were patients with suspected atypical AFL or prior ablation. Non-AFL arrhythmias were excluded by a minimum of three 12-lead ECGs performed on separate occasions, or ambulatory Holter monitoring prior to enrolment. The full inclusion and exclusion criteria have been previously published [11].

Patients were randomised in a 1:1 ratio to either cryoballoon PVI (intervention arm) or radiofrequency (RF) CTI ablation (control arm). All patients had an ILR (Medtronic LINQ) implanted immediately following ablation. After a 12-month review, patients were asked if they would re-consent for a further 2 years of follow-up, with study visits at 24 and 36 months.

The study was approved by ethical review committees in both countries (UK and Switzerland) and was registered on ClinicalTrials.gov (NCT03401099).

### Ablation procedures

Detailed descriptions of the ablation procedures have been previously published [1, 11]. Briefly, PVI was undertaken with the Artic Front Advance (Medtronic) cryoballoon targeting entrance and exit block in all PVs, without targeted non-PV trigger ablation. CTI ablation was performed with an RF catheter, with the exact equipment and approach at the operator’s discretion, targeting an endpoint of a bidirectional CTI conduction block.

### Primary, secondary and safety outcomes

The primary outcome was the time-to-first recurrence of symptomatic atrial arrhythmia lasting > 30 s (i.e., recurrence of AFL or new onset of AT or AF), following a 4-week post-ablation blanking period. A 4-week blanking period was chosen to ensure consistency between the two randomised groups, and also because arrhythmia occurring beyond 4 weeks has been shown to be associated with recovery in pulmonary vein conduction [12]. Antiarrhythmic drugs were discontinued after the blanking period but could be restarted at the clinician’s discretion. Secondary outcomes included time to first AF episode lasting ≥ 2 min (minimum duration detectable by the ILR), time-to-first-episode of AFL or atrial tachycardia (of any duration), atrial arrhythmia burden on ILR (to end of follow-up, or to time of cardioversion or repeat ablation if this occurred) and the need for redo ablation or cardioversion.

The primary safety outcome was a composite of death, stroke, transient ischaemic attack (TIA), tamponade requiring pericardiocentesis, atrio-oesophageal fistula, an implant of a permanent pacemaker, a vascular injury requiring intervention or delaying discharge, or persistent phrenic nerve palsy (> 24 h). Secondary safety outcomes included the individual components of the composite outcome and minor vascular complications, unplanned hospitalisation, pericarditis or pericardial effusion or myocardial infarction.

### Statistical analysis

The power calculation was described previously and was estimated for a 12-month follow-up [11]; hence, this extended follow-up provides additional data but without a formal power calculation.

Outcomes were analysed by the intention-to-treat principle. Continuous variables were described as mean ± standard deviation or median (25th–75th quartile) and tested for differences using *t*-tests or non-parametric equivalents depending upon the distribution. Categorical variables were described as counts and percentages and tested using Fisher’s exact test. Kaplan–Meier plots and Cox proportional hazard regression models were used to describe time-to-event outcomes. A two-sided *p*-value of < 0.05 was considered statistically significant. All statistical analysis was performed in R (v 4.3.0; R Foundation).

## Results

### Patient cohorts and characteristics

From August 2018 to March 2020, a total of 113 patients were enrolled across 11 sites, nine in the United Kingdom and two in Switzerland. The original target was 130 patients; however, recruitment had to be terminated early at 87% of target recruitment due to the outbreak of the COVID-19 pandemic.

**Fig. 1:**
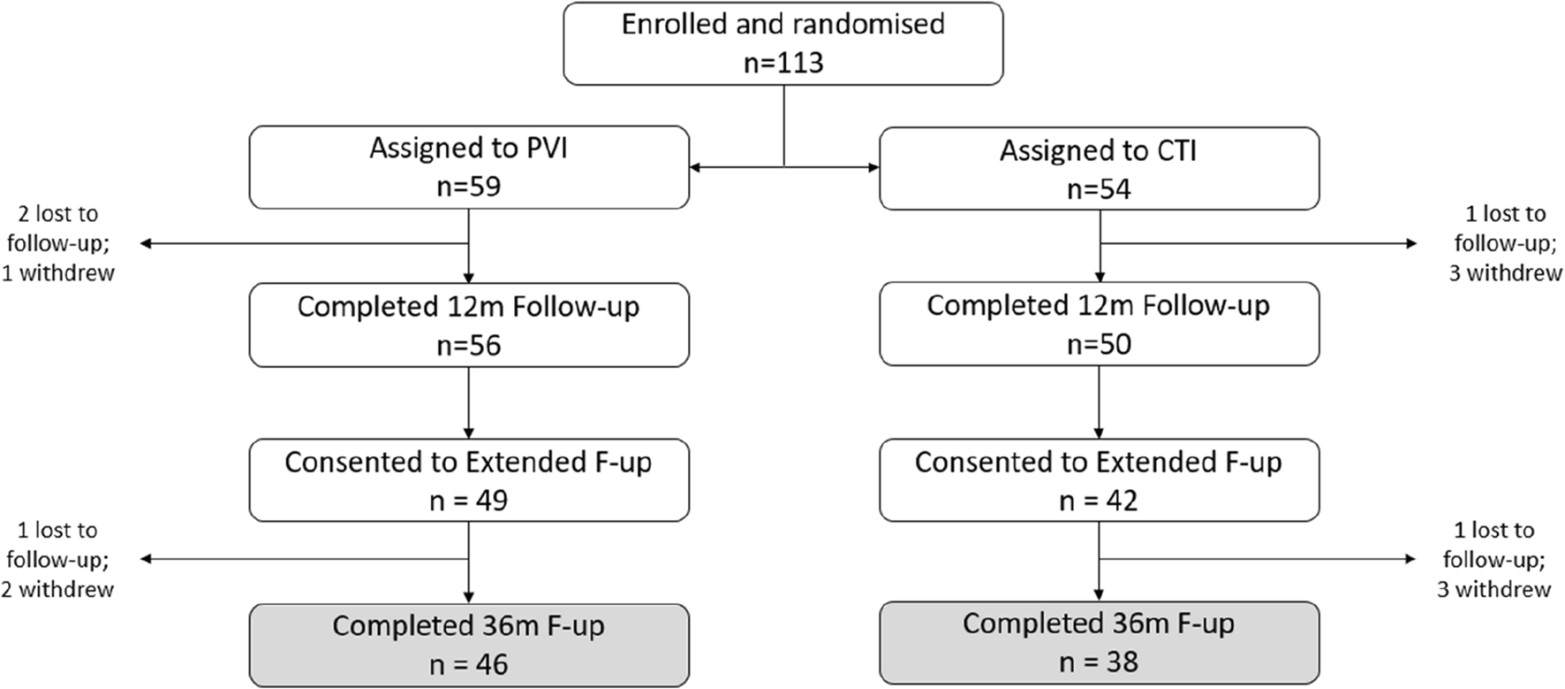
Trial consort diagram. CTI cavotricuspid isthmus, PVI pulmonary vein isolation

Overall, 91 patients re-consented at 12 months for extended follow-up—49 in the PVI arm and 42 in the CTI arm. A post-randomisation flow is shown in Fig. 1. Baseline patient characteristics per arm are shown in Table 1. Table 2 shows the demographics of those who reconsented for extended follow-up.

**Table 1:** Demographic data at baseline recruitment

Baseline data	PVI (*n* = 59)	CTI ablation (*n* = 54)
Age (years), mean ± SD	66 ± 9	67 ± 11
Male sex, *n* (%)	52 (88.1)	46 (85.2)
BMI (kg/m^2^), mean (SD)	29.6 ± 4.5	27.9 ± 4.08
Smoking status, *n* (%)		
Current	3 (5.2)	6 (11.3)
Past	29 (50.0)	21 (39.6)
Never	26 (44.8)	26 (49.1)
Alcohol score*, mean ± SD	4.6 ± 3.1	3.9 ± 3.0
Months since flutter diagnosis, median (IQR)	6 (3–12)	8 (3–19.5)
Previous cardioversion, *n* (%)	28 (47.5)	27 (50.0)
LA diameter (cm), mean ± SD	4.0 ± 0.6	4.2 ± 0.6
LVEF (%), mean ± SD	54.2 ± 8.3	54.7 ± 10.2
Comorbidities, *n* (%)		
Hypertension	17 (28.8)	17 (31.5)
Diabetes	10 (16.9)	2 (3.7)
Prior ACS	4 (6.8)	1 (1.9)
Prior PCI	4 (6.8)	2 (3.7)
Prior CABG	5 (8.5)	0 (0.0)
Heart failure	1 (1.7)	2 (3.7)
Prior stroke, TIA, or systemic embolism	7 (11.9)	5 (9.3)
CHA_2_DS_2_Vasc score, mean ± SD	1.6 ± 1.4	1.7 ± 1.3
Medication, *n* (%)		
Beta blocker	40 (67.8)	42 (77.8)
Calcium channel blocker	10 (16.9)	3 (5.6)
ACE inhibitor/ARB	12 (20.3)	13 (24.1)
Class I antiarrhythmic	3 (5.1)	1 (1.9)
Class III antiarrhythmic	3 (5.1)	5 (9.3)
Digoxin	3 (5.1)	7 (13.0)

Alcohol score—AUDIT-C questionnaire ranging from 0 (lowest risk) to 12 (highest risk)

*ACE* angiotensin-converting enzyme, *ACS* acute coronary syndrome, *ARB* angiotensin 2 receptor blocker, BMI body mass index, *CABG* coronary artery bypass graft, *IQR* interquartile range, *LA* left atrium, *LV* left ventricle, *PCI* percutaneous coronary intervention, *SD* standard deviation, *TIA* transient ischaemic attack

**Table 2:** Demographic data for patients who reconsented for extended follow-up (*n* = 90)

Baseline data	PVI (*n* = 49)	CTI ablation (*n* = 42)	*P*-value
Age (years), mean ± SD	65.1 ± 9.4	68.4 ± 9.2	0.104
Male sex, *n* (%)	43 (87.8)	35 (83.3)	0.565
BMI (kg/m^2^), mean (SD)	29.5 ± 4.4	27.8 ± 4.0	0.059
Smoking status, *n* (%)			0.127
Current	1 (2.1)	5 (12.2)	
Past	25 (52.1)	16 (39.0)	
Never	22 (45.8)	20 (48.8)	
Alcohol score*, mean ± SD	4.5 ± 3.2	4.4 ± 3.0	0.855
Months since flutter diagnosis, median (IQR)	6 (3–12)	10.5 (3–20)	0.210
Previous cardioversion, *n* (%)	25 (51.0)	23 (54.8)	0.834
LA diameter (cm), mean ± SD	4.0 ± 0.6	4.2 ± 0.6	0.183
LVEF (%), mean ± SD	55.5 ± 7.8	55.5 ± 9.9	0.975
Comorbidities, *n* (%)			
Hypertension	11 (22.4)	13 (31.0)	0.475
Diabetes	7 (14.3)	1 (2.4)	0.065
Prior ACS	4 (8.2)	1 (2.4)	0.369
Prior PCI	4 (8.2)	2 (4.8)	0.683
Prior CABG	4 (8.2)	-	0.121
Heart failure	1 (2.0)	2 (4.8)	0.593
Prior stroke, TIA, or systemic embolism	4 (8.2)	4 (9.5)	> 0.999
CHA_2_DS_2_Vasc score, mean ± SD	1.4 ± 1.4	1.7 ± 1.3	0.194
Medication, *n* (%)			
Beta blocker	33 (67.3)	32 (76.2)	0.486
Calcium channel blocker	8 (16.3)	3 (7.1)	0.213
ACE inhibitor/ARB	9 (18.4)	10 (23.8)	0.609
Class I antiarrhythmic	3 (6.1)	1 (2.4)	0.621
Class III antiarrhythmic	2 (4.1)	3 (7.1)	0.659
Digoxin	2 (4.1)	3 (7.1)	0.659

Alcohol score—AUDIT-C questionnaire ranging from 0 (lowest risk) to 12 (highest risk)

*ACE* angiotensin-converting enzyme, *ACS* acute coronary syndrome, *ARB* angiotensin 2 receptor blocker, *BMI* body mass index, *CABG* coronary artery bypass graft, *IQR* interquartile range, *LA* left atrium, *LV* left ventricle, *PCI* percutaneous coronary intervention, *SD* standard deviation, *TIA* transient ischaemic attack

### Primary outcome

**Fig. 2:**
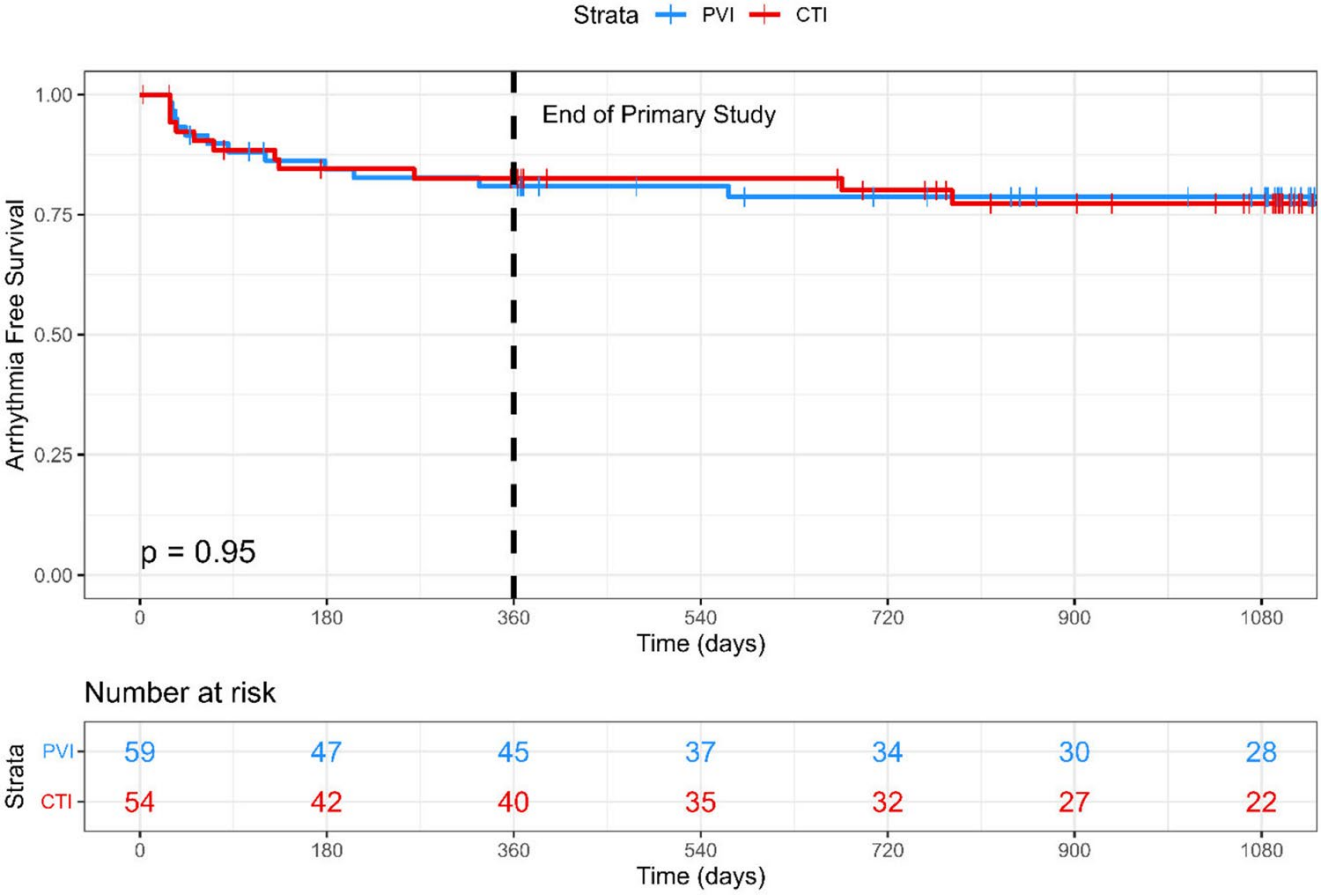
Kaplan–Meier plot for the primary outcome of symptomatic arrhythmia recurrence over the 36-month follow-up

Across the 36-month follow-up, 23 (12 PVI, 11 CTI) patients met the primary outcome. There was no difference in time-to-event between groups (HR 0.97; 95% CI 0.43–2.20; *p* = 0.944; Fig. 2). Using Kaplan–Meier analysis, the estimated arrhythmia freedom at 12 months was 80.9% (95% CI 71.3–93.6%) in the PVI group and 82.5% (95% CI 72.8–93.6%) in the CTI group. The estimated 36-month arrhythmia freedom was 78.7% (95% CI 68.6–90.2%) in the PVI group and 77.3% (95% CI 66.2–90.3%) in the CTI group.

Of those who met the primary outcome, episodes adjudicated as AF occurred less frequently in the PVI group (4/12 [33%] vs 9/11 [82%]; *p* = 0.036) whilst episodes adjudicated as AFL tended to occur more frequently in the PVI group (7/12 [58%] vs 2/11 [18%]; *p* = 0.089). Atrial tachycardia occurred in a single patient (8%) in the PVI group only.

### Secondary outcomes

**Fig. 3:**
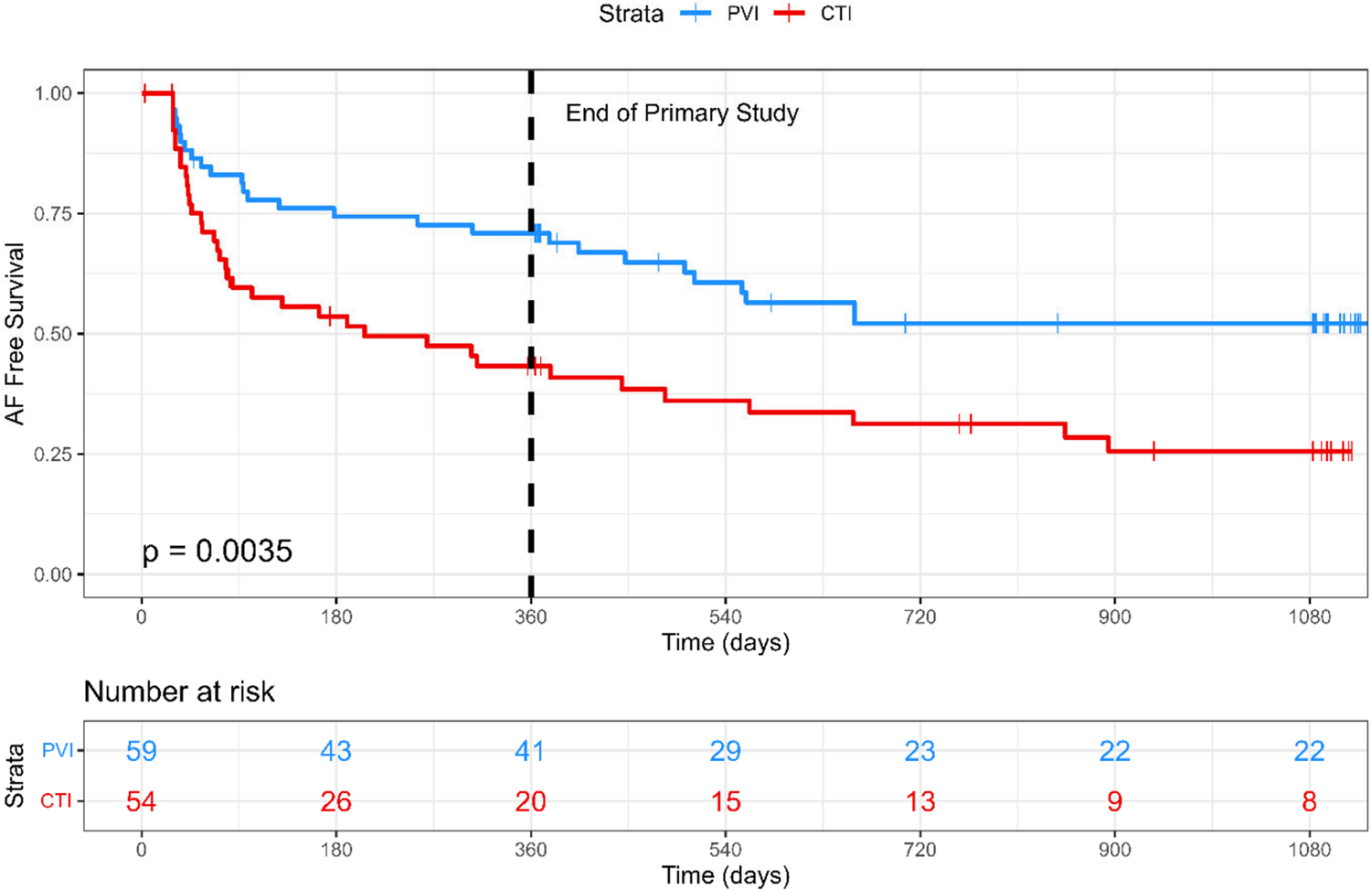
Kaplan–Meier plot for the secondary outcome of AF occurrence ≥ 2 min over the 36-month follow-up

Significantly fewer patients in the PVI arm experienced an episode of AF ≥ 2 min compared with the CTI arm (HR 0.48; 95% CI 0.29–0.79; *p* = 0.004; Fig. 3).

**Fig. 4:**
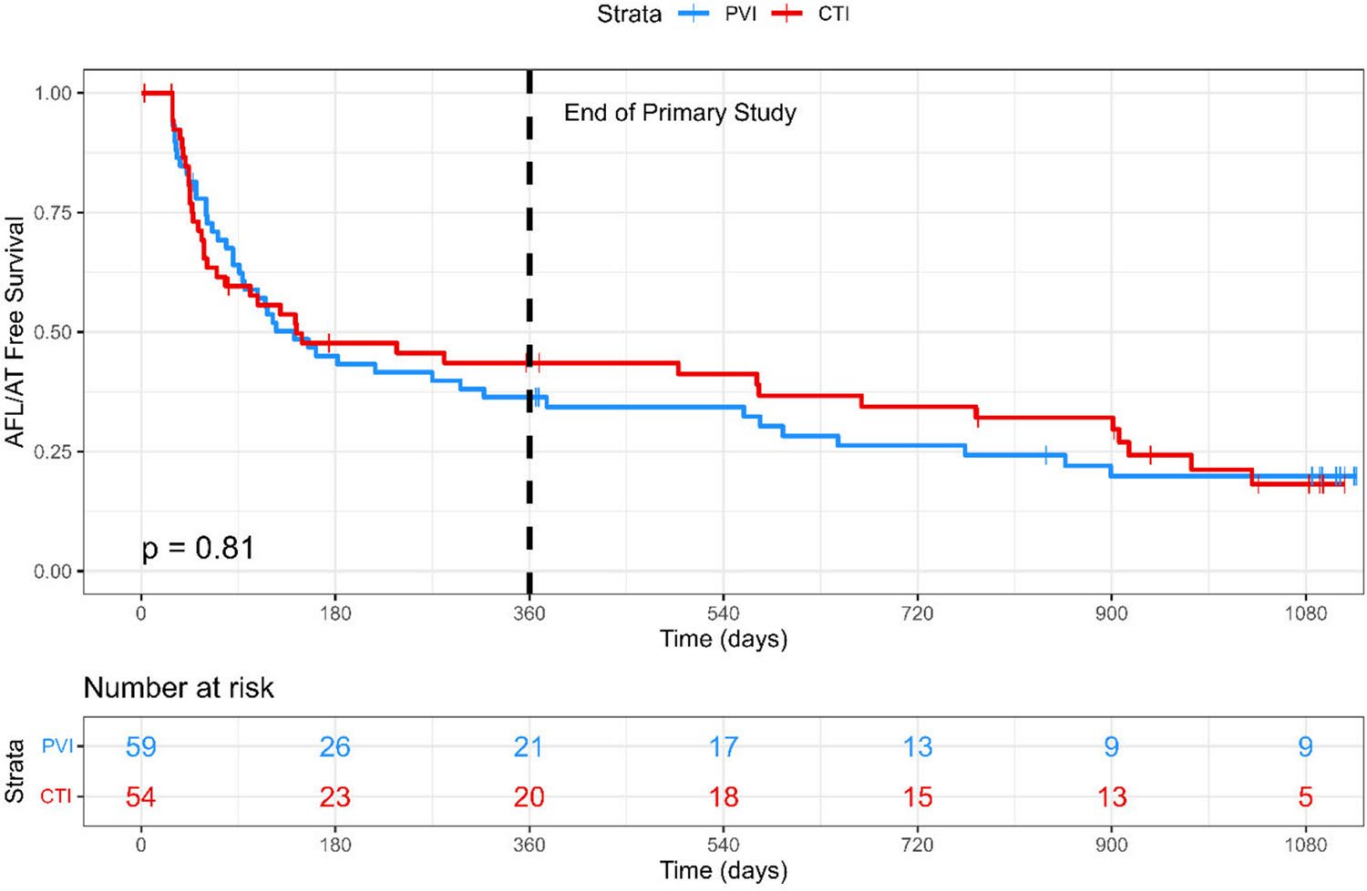
Kaplan–Meier plot for the secondary outcome of AFL/AT occurrence of any duration over the 36-month follow-up

Time to AFL or AT of any duration did not differ between the groups (HR 1.06; 95% CI 0.69–1.62; *p* = 0.806; Fig. 4). The median arrhythmia burden was also similar between groups (PVI 0.01% vs CTI 0.06%; *p* = 0.131).

**Fig. 5:**
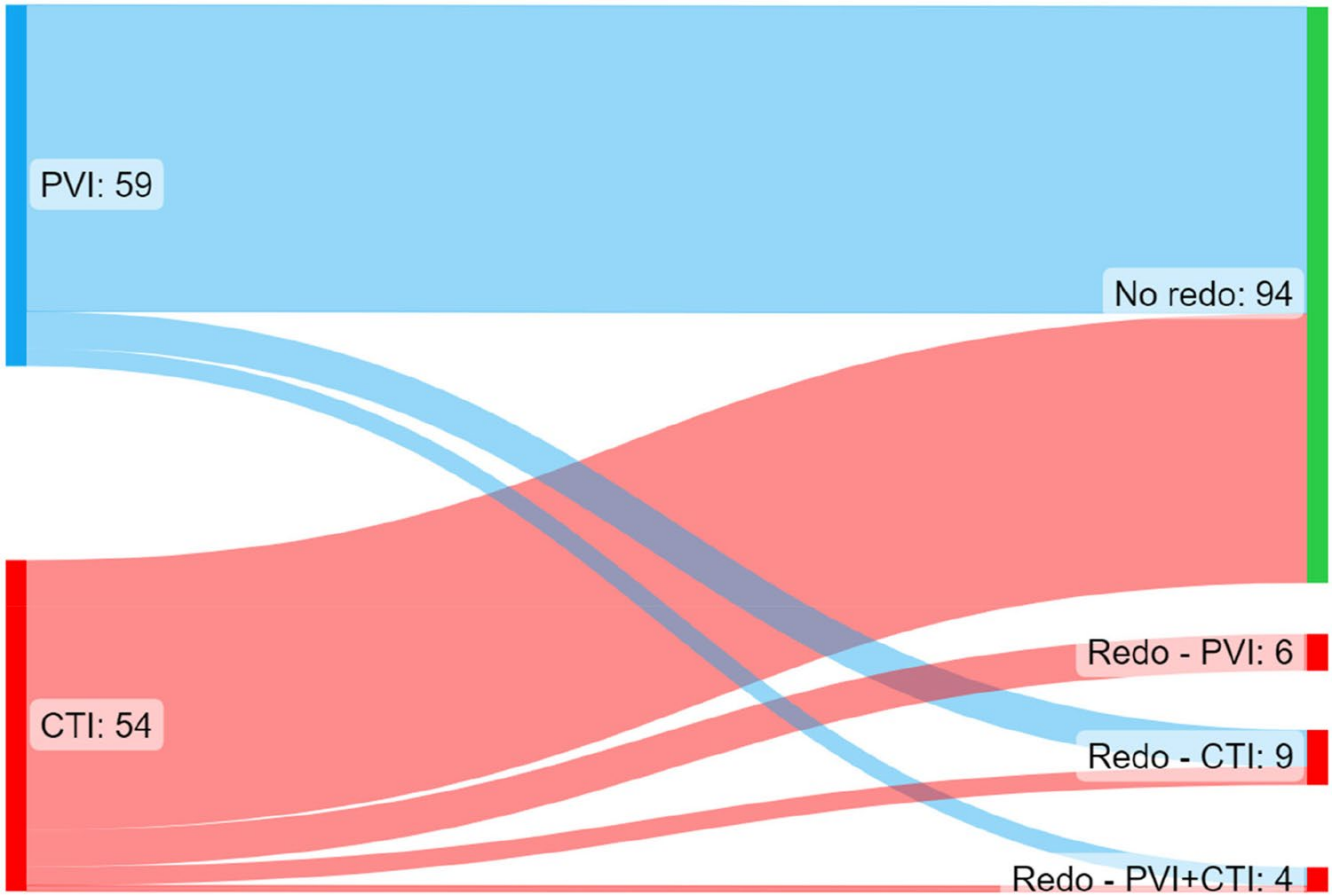
Sankey plot showing redo ablations across 36-month follow-up

Electrical cardioversion beyond the blanking period was required in 8 PVI patients vs 4 CTI patients (13.6% vs 7.4%; *p* = 0.367). Redo ablation was required in 9 PVI patients vs 10 CTI patients (15.3% vs 18.5%; *p* = 0.802). In the PVI arm, all 9 redo ablations involved the creation of a CTI line, though 3 of these patients additionally had redo PVI. In the CTI group, 7 redo patients underwent PVI, one of whom additionally had redo CTI ablation; the remaining 3 underwent redo CTI only. These figures are summarised in Fig. 5.

Including only those who completed each follow-up timepoint, antiarrhythmic drugs were being used by 1 patient (0.9%) at 12 months, 1 patient (1.1%) at 24 months and 2 patients (2.4%) at 36 months.

### Safety outcomes

The primary composite safety outcome occurred in 5 patients in the PVI arm and 6 patients in the CTI arm (8.5% vs 11.1%; *p* = 0.755). Overall, complications were not statistically different between the two arms (Table 3).

**Table 3:** Complications and adverse events

Complications, *n* (%)	PVI (*n* = 59)	CTI ablation (*n* = 54)	*P*-value
Composite safety endpoint	5 (8.5)	6 (11.1)	0.755
Groin haematoma	2 (3.4)		0.497
All-cause hospitalisation*	18 (30.5)	12 (22.2)	0.395
Pericarditis			
Pericardial effusion	1 (1.7)		> 0.999
Phrenic nerve palsy			
Requirement for pacemaker	1 (1.7)	5 (9.3)	0.102
Stroke or TIA			
Myocardial infarction	1 (1.7)		> 0.999
Atrio-oesophageal fistula			
All-cause death	3 (5.1)	1 (1.9)	0.620

*CTI* cavotricuspid isthmus ablation, *PVI* pulmonary vein isolation, *TIA* transient ischaemic attack

^*^All-cause hospitalisation is described here as a binary outcome, i.e., a patient either had a hospitalisation during follow-up or did not. Further details about all-cause hospitalisation is given in the text

All-cause hospitalisation was required in 18 (30.5%) PVI patients vs 12 (22.2%) CTI patients (*p* = 0.395). Of these patients, the majority (*n* = 23) had only a single hospitalisation reported across follow-up. Smaller numbers had multiple hospitalisations: 3 patients had 2 hospitalisations, 2 patients had 3 hospitalisations and 2 patients had 5 hospitalisations.

Six patients required pacemaker implants, though none of these were considered directly related to the ablation procedure. Four pacemakers were implanted due to incidental bradyarrhythmia found on the ILR (symptomatic pauses in most cases, complete atrioventricular block in one case > 12 months post-ablation). One pacemaker was implanted as part of a pace-and-ablate strategy, and one was implanted to manage a complete atrioventricular block complicating a different (cardiac surgical) procedure.

## Discussion

The results of the CRAFT extension study show that in patients presenting with isolated typical AFL with no previously documented AF, cryoballoon PVI resulted in a similar reduction in symptomatic atrial arrhythmia as compared to RF CTI ablation. Importantly, the incidence of AF was significantly reduced with an upfront PVI approach, without creating any significant safety signals. The extended follow-up of 3 years showed that whilst the Kaplan–Meier curves for AF occurrence diverged early, this difference was preserved over time. This is an important observation and refutes a potential concern with the 12-month results [11] that a PVI-only approach may have merely delayed the development of arrhythmia.

### Atrial arrhythmia—triggers vs mechanisms

As described earlier, it is theorised that all patients with AFL have underlying PV triggers, and those susceptible to developing an intercaval functional line of block may organise into a typical flutter circuit [2–4]. The results of the CRAFT study demonstrate that addressing the arrhythmia trigger, without targeting the mechanism, can result in similar arrhythmia freedom to the elimination of the mechanism itself. Indeed, 78.8% (Kaplan–Meier estimate) of patients in the PVI group stayed free of symptomatic arrhythmia recurrence in spite of no CTI ablation having been performed, giving support to this hypothesis. It should be acknowledged, however, that symptomatic recurrence can be underestimated when using ILR follow-up, as it relies upon patient reporting.

Such an approach may be beneficial in selected cases, with shared decision-making. For example, a patient with isolated AFL, but with comorbidities and left atrial enlargement, may be more likely to re-present with AF and may benefit from early elimination of PV triggers. Whilst it can be argued that PVI is a higher-risk procedure—due to the necessity for transseptal puncture and left atrial access—we did not find a difference in safety outcomes (accepting that our study was not powered to detect such rare events). Furthermore, a recently published comprehensive review has shown that AF ablation has become an increasingly safe procedure over time [13], which may support a lower threshold for considering a trigger-based PVI strategy.

Although not explicitly studied here, in those patients where a PVI approach to AFL is considered, it may be prudent to perform both PVI and CTI ablation in the same procedure. This is because AFL recurrences do happen, particularly after a single PVI procedure; thus, the patient may gain maximal benefit from targeting both trigger *and* mechanism.

### Arrhythmia recurrences

Although recurrences and redo procedures were relatively infrequent, it is notable that PVI patients were numerically more likely to experience AFL and require CTI ablation, and CTI patients were more likely to experience AF and require PVI. Aside from highlighting the benefits of targeting both, as mentioned above, it is important to consider the mechanisms at play when arrhythmia recurs. We did not perform routine re-mapping of the left atrium to check for PV reconnections in this study. It is therefore possible that PVI patients presenting with recurrent AFL simply had a PV reconnection, thus facilitating re-initiation of the unablated CTI mechanism. Hence, robust PVI may, in theory, eliminate typical AFL. However, given the well-known difficulty in achieving durable PVI at present, as well as the potential for non-PV triggers, it again stands to reason that CTI ablation should be recommended for the majority. This is in line with prior studies assessing the combined procedure versus CTI ablation alone [14–20]. Routinely ablating the CTI in patients with isolated AF who have not demonstrated clinical AFL is not recommended [21–25].

It is notable that a large percentage of patients have short bursts or asymptomatic atrial tachyarrhythmia, as shown in Fig. 4. Atrial high-rate episodes have recently been discussed as pre-cursors to AF [26]—in our study, ablation reduced the amount of sustained arrhythmia, but these episodes may still be present as shown by ILR interrogation. These sub-clinical episodes may reflect the ongoing presence of arrhythmogenic substrate despite symptomatic resolution. This may be important in terms of decision-making around long-term anticoagulation. Alternatively, these may be unimportant features which are only detected due to continuous ILR monitoring. The recent NOAH-AFNET 6 study found that patients with incidentally detected atrial high-rate episodes did not benefit from oral anticoagulation [26]; however, these patients had not been diagnosed with AF or AFL, nor had they undergone prior catheter ablation. Further research would be beneficial.

### Current practice and future implications

The CRAFT trial provides supportive evidence for considering PV trigger elimination in patients presenting with isolated typical AFL, as well as providing insight into the relationship between mechanisms and triggers of atrial arrhythmia. This may support decision-making in patients at high risk of presenting with subsequent AF. Equally, our results may give reassurance in the setting where bidirectional CTI block cannot be achieved but PVI is feasible.

The field of cardiac electrophysiology has evolved considerably over time [13], especially since the CRAFT study began, particularly with the recent advent of pulsed-field ablation (PFA). Future work may include assessing the effect of PFA-based PVI on AFL. Ongoing work to understand the optimal approach to atrial arrhythmia ablation—be it trigger or mechanism ablation—will be crucial in the years to come.

## Limitations

Our study has several limitations. Firstly, our study was underpowered to detect the primary outcome due to (a) early termination due to the COVID-19 pandemic and (b) a lower-than-expected event rate in the control arm. This is somewhat ameliorated by longer-term follow-up in this extension study, though also affected by the degree of drop-out after 12 months. Secondly, we implanted ILRs at the time of ablation, which precluded (a) the assessment of arrhythmia burden prior to ablation and (b) more robust confirmation of the absence of pre-existing AF. Thirdly, though ILRs are considered the gold standard for arrhythmia studies, they provide only a single ECG channel, are prone to artefact and can be less accurate for detecting regular arrhythmias such as AFL. Reporting of symptomatic arrhythmia also relied upon patient adherence (pressing their device button to record a symptomatic episode)—although all reported episodes were analysed, non-adherence may result in under-detection of symptomatic events. Sites were not blinded to ILR findings, which may affect the generalisability of findings; however, as ILR findings were only given in the event of a direct request or for safety reasons (e.g., ventricular arrhythmias or significant bradycardia), most patients were not actively treated in direct response to ILR findings. Additionally, patients were not blinded to treatment allocation; although, the experience of an ablation procedure to a patient will have been generally similar between arms. Finally, although our primary outcome showed similar outcomes between arms, our study was not designed, nor powered, for non-inferiority, and thus, equivalence cannot be conclusively demonstrated.

## Conclusion

Cryoballoon PVI provides similar long-term symptomatic arrhythmia suppression as RF CTI ablation in patients presenting with isolated typical AFL, without adversely affecting safety. When managing such patients, the impact of PV triggers, and the likelihood of subsequent AF should be considered.

## Data Availability

Data can be made available upon reasonable request.
